# Transcriptome of American Oysters, *Crassostrea virginica,* in Response to Bacterial Challenge: Insights into Potential Mechanisms of Disease Resistance

**DOI:** 10.1371/journal.pone.0105097

**Published:** 2014-08-14

**Authors:** Ian C. McDowell, Chamilani Nikapitiya, Derek Aguiar, Christopher E. Lane, Sorin Istrail, Marta Gomez-Chiarri

**Affiliations:** 1 College of the Environment and Life Sciences, University of Rhode Island, Kingston, Rhode Island, United States of America; 2 Department of Computer Science and Center for Computational Molecular Biology, Brown University, Providence, Rhode Island, United States of America; Institut Pasteur Paris, France

## Abstract

The American oyster *Crassostrea virginica*, an ecologically and economically important estuarine organism, can suffer high mortalities in areas in the Northeast United States due to Roseovarius Oyster Disease (ROD), caused by the gram-negative bacterial pathogen *Roseovarius crassostreae*. The goals of this research were to provide insights into: 1) the responses of American oysters to *R. crassostreae,* and 2) potential mechanisms of resistance or susceptibility to ROD. The responses of oysters to bacterial challenge were characterized by exposing oysters from ROD-resistant and susceptible families to *R. crassostreae*, followed by high-throughput sequencing of cDNA samples from various timepoints after disease challenge. Sequence data was assembled into a reference transcriptome and analyzed through differential gene expression and functional enrichment to uncover genes and processes potentially involved in responses to ROD in the American oyster. While susceptible oysters experienced constant levels of mortality when challenged with *R. crassostreae,* resistant oysters showed levels of mortality similar to non-challenged oysters. Oysters exposed to *R. crassostreae* showed differential expression of transcripts involved in immune recognition, signaling, protease inhibition, detoxification, and apoptosis. Transcripts involved in metabolism were enriched in susceptible oysters, suggesting that bacterial infection places a large metabolic demand on these oysters. Transcripts differentially expressed in resistant oysters in response to infection included the immune modulators IL-17 and arginase, as well as several genes involved in extracellular matrix remodeling. The identification of potential genes and processes responsible for defense against *R. crassostreae* in the American oyster provides insights into potential mechanisms of disease resistance.

## Introduction

The American or eastern oyster *Crassostrea virginica* is an estuarine molluscan bivalve species fished and cultured from Texas, USA to New Brunswick, Canada. Oyster production is an important sector of United States agriculture and the American oyster was estimated in 2012 to have a farm gate value of $104 million in the United States [1]. Ecologically, the American oyster provides biogenic habitat and filters large quantities of plankton, having a great impact on the coastal ecosystems it inhabits [2], [3]. Several oyster diseases, both protozoan and bacterial, have expanded in range and increased in severity during the latter half of the twentieth century, often causing staggering losses [4]. Juvenile or Roseovarius Oyster Disease (ROD), an emerging disease caused by the gram-negative bacterium *Roseovarius crassostreae*, was first reported in 1988 and presently affects oysters from the Long Island Sound north to Maine [5], [6]. As high as 90–100% of oyster juveniles in a farm may succumb to this disease during mortality events that often coincide with peak summer water temperatures. Gross clinical signs include uneven shell margins, soft tissue emaciation, and conchiolin depositions (a mix of shell material and organic molecules) on the inner shell surfaces [5]–[7].

The host-pathogen interactions between *C. virginica* and *R. crassostreae* are poorly understood. This extracellular bacterial pathogen colonizes the oyster’s inner shell surface before lesions develop in the epithelial mantle. Colonization of the inner side of the oyster shell by *R. crassostreae* likely stimulates oysters to deposit conchiolin [8]. It has been hypothesized that smaller juvenile oysters (<25 mm in shell length) are most susceptible to ROD because they lack adequate metabolic resources to fuel immune responses, including conchiolin deposition, leading to emaciation [5], [6], [8]. *Roseovarius crassostreae* may produce a toxin with ciliostatic activity [9] and extracellular products from *R. crassostreae* have a cytotoxic effect on oyster hemocytes that cannot be solely attributed to lipopolysaccharide (LPS), a component of the membrane of gram-negative bacteria [10].

Traditional selective breeding practices have led to the production of ROD-resistant oysters [11], [12], but the genetic basis of resistance is presently unknown. The identification of potential genes and pathways responsible for an effective host defense response in the American oyster to *R. crassostreae* is important not only to provide a basis for enhanced breeding techniques [13]–[15], but also advances the understanding of immunity in a member of Lophotrochozoa, a superphylum that has been poorly represented among genomic and transcriptomic datasets until recently *e.g*. [16]–[19]. Invertebrate hosts lack the classical adaptive immune system, yet they successfully combat widely varied types of microbes and parasites. To mount effective and flexible defense responses to diverse pathogens, invertebrate hosts have developed diversified repertoires of receptors, regulators, and/or effectors including Toll-like receptors (TLRs), fibrinogen-related proteins (FREPs), scavenger receptor cysteine-rich (SRCRs), and antimicrobial proteins, as well as many other molecules involved in the key processes of agglutination, phagocytosis, and encapsulation [20]–[22].

In order to identify genes and processes potentially involved in: 1) the responses of American oysters to challenge with the bacterial pathogen *R. crassostreae,* and 2) potential mechanisms of resistance or susceptibility to ROD, cDNA sequences from ROD-resistant and susceptible families of oysters exposed to the bacterial pathogen *Roseovarius crassostreae* were assembled into a reference transcriptome. A targeted differential gene expression analysis, followed by evaluation of functional categories enriched among differentially expressed genes, were used to identify genes and processes involved in the response of oysters exposured to *R. crassostreae*. This targeted analysis was also used to identify a list of genes and molecular processes potentially involved in resistance/susceptibility to ROD.

## Materials and Methods

### Bacterial challenge of American oysters

Juvenile American oysters from 2 families with known differential susceptibility to ROD (F3L and GX09) were kindly provided by X. Guo (Haskin Shellfish Research Laboratory, Rutgers University). Susceptible F3L oysters were F_3_ generation progeny from a single pair mating of a female oyster from the Rutgers NEH (Northeastern High-survival) line [23] and a male oyster from Louisiana (LA). Resistant GX09 (GX) oysters were an F_3_ generation containing germline material from the NEH, DBH (Delaware Bay High-survival line), LA, and the ROD-resistant lines UMFS (University of Maine Flowers Select) and FMF (Frank M. Flowers) lines [11], [12]. Oysters (10–15 mm in shell length) were labeled on the outside of the shell using non-toxic paint (to distinguish each family) and placed into two replicate 250 l tanks with filtered sterile seawater (FSSW) for bacterial challenge (experimental groups GX and F3L, about 120 oysters per family per tank). Additional groups of 50 (F3L) and 2×50 (GX) oysters were kept in 50 l tanks as unchallenged controls (CGX and CF3L). Oysters were acclimated during a period of 2 weeks to experimental conditions (salinity 28–30‰, temperature 19°C). Oysters in the challenge tanks were exposed to *R. crassostreae*, strain CV919-312^T^ [7] by addition of bacteria to the tank at a final concentration of 7.5×10^6^ colony forming units (CFU) ml^−1^ (day 0 of challenge). Oysters were fed Instant Algae (Reed Mariculture) every other day and water was partially changed (50%) weekly. Oysters were monitored weekly for 93 days for mortalities and for the presence of clinical signs of ROD (uneven valves and conchiolin deposits in shells of dead oysters). Infection by *R. crassostreae* was confirmed by PCR [24].

### Sample collection, cDNA preparation, and sequencing

Oyster whole body tissue was collected from 15 randomly sampled oysters each from CGX, CF3L (unchallenged controls), GX, and F3L (challenged resistant and susceptible oysters) at days 1, 5, 15, and 30 following challenge and stored in RNAlater until time of RNA isolation. All RNA molecules >200 nucleotides were purified using Qiagen RNAeasy Mini Kit. Samples were checked for RNA purity using a Nanodrop 8000 spectrophotometer and a subset of the extracts were checked using an Agilent 2100 Bioanalyzer. Due to limitations in funding, control non-challenged susceptible (CF3L) oysters were not included in the sequencing analysis. Equal amounts of total RNA from 5 oysters from each treatment and time point (excluding C3FL) were pooled for a total of 12 experimental samples (3 treatments×4 time points). Samples of RNA were selectively enriched for poly-A containing mRNA and cDNA libraries for sequencing were prepared using the Illumina mRNA-Seq-8 Sample Prep Kit. The cDNA libraries were sequenced on the Illumina GAIIx platform (1 lane per sample for a total of 12 lanes, Genome Quebec, Canada).

### Read processing and de novo assembly

Raw sequencing reads of 108 bp from all lanes (SRP042090) were pooled, processed, and filtered for contamination of mitochondrial and ribosomal sequences by mapping to all *Crassostrea* spp. rRNA and mtDNA in NCBI Genbank database. Reads were filtered for vector sequences by mapping to Univec (ftp://ftp.ncbi.nih.gov/pub/univec) using bowtie2 [25]. Low-complexity artifacts were removed, and Illumina adapters and the 5′-ends of reads were trimmed using the fastx-toolkit (http://hannonlab.cshl.edu/fastx_toolkit/). Adaptor trimming was performed on reads using the btrim software package [26]. Reads less than 20 bp in length were discarded. Processed transcriptome reads from the 12 lanes were assembled into a reference transcriptome using Trinity (release 20111126) with default options [27]. Only those assembled contigs ≥200 bp were retained. Transcriptome contigs were compared to the RefSeq protein database [28]. A custom python script, created by L. Dong (Brown University), was used to parse BLAST output and identify possible contaminants. Contigs that had all top blast hits (a maximum of 10) with associated e-value≤1e-06 to proteins from Archaebacteria, Bacteria, or Protozoa were discarded. Additional mitochondrial and ribosomal contaminants were identified and discarded through text searching of BLAST results. Phage integrase sequences were identified and discarded by comparing the transcripts to Pfam_A using Pfam scan (version 1.3) and HMMER (version 3.0) with hits retained where e-value≤1e-05 [29], [30]. DNA transposons were identified and discarded using RepeatMasker [31].

### Differential Gene Expression

Reads from individual treatment-days samples (e.g. GX-1d) were aligned to the reference transcriptome using bowtie [32] with parameters “-v 3–a –best –strata,” such that 3 mismatches were allowed per read to account for the high rate of polymorphism in oysters [33]. Transcript abundances in reads per kilobase per million reads mapped (RPKM) were estimated using RSEM (RNA-Seq by Expectation Maximization) through the Trinity plug-in, run_RSEM.pl. [27], [34], [35]. To reduce bias from differential sequencing depth across lanes, the trimmed mean of M values (TMM) method was used to calculate normalization factors for each lane [36]. Only those contigs with at least 1 count-per-million in at least 2 samples were tested for differential expression.

In order to identify general patterns of variation driving differences between treatment groups, two analyses were performed in the R programming environment [37] using the Z-score centered log_2_-transformed RPKM for each transcript in each of the treatment groups: a) a principal components analysis (PCA); and b) a heatmap analysis. For the heatmap analysis, transcripts were hierarchically clustered (Euclidean distance, complete linkage) using the fastcluster [38] package. Results were visualized using gplots [39]. Based on the results from the mortality curves and the principal components and heatmap analyses, differential gene expression analysis was performed by comparing read abundances for contigs in each of the samples to read abundances in a control pool (CGX 15 and 30 d, see results for rationale) using edgeR [40]. Significance values yielded by hypergeometric test were adjusted using the False Discovery Rate (FDR) correction and a contig was considered differentially expressed (DE) if it had an FDR-adjusted p-value≤0.05 [41].

### Annotation and Functional Enrichment

Transcriptome contigs were compared to the NCBI protein non-redundant (NR) database using BLASTx [42]. Hits with e-value≤1e-6 were retained. Gene Ontology (GO) terms were mapped to the best BLASTx hits for each contig using the Blast2GO pipeline (version 2.3.5) [43]. In order to identify which functional categories were enriched within differentially expressed transcripts for the selected treatment groups, functional enrichment was performed using the R package topGO by comparing the numbers of GO terms associated with annotations of differentially expressed transcripts within each selected treatment to the numbers of terms associated with all transcripts not differentially expressed [44]. Fisher’s exact test was used to determine significance of enrichment of each GO term, with Bonferroni-adjusted p-values≤0.05 taken as significant. Functionally enriched GO terms were visualized in semantic space using SimRel functional similarity measure [45] and the REViGO online visualization tool [46] modified with the R package ggplot2 [47] (scripts available upon request).

## Results

### Oyster survival in response to bacterial challenge

**Figure 1:**
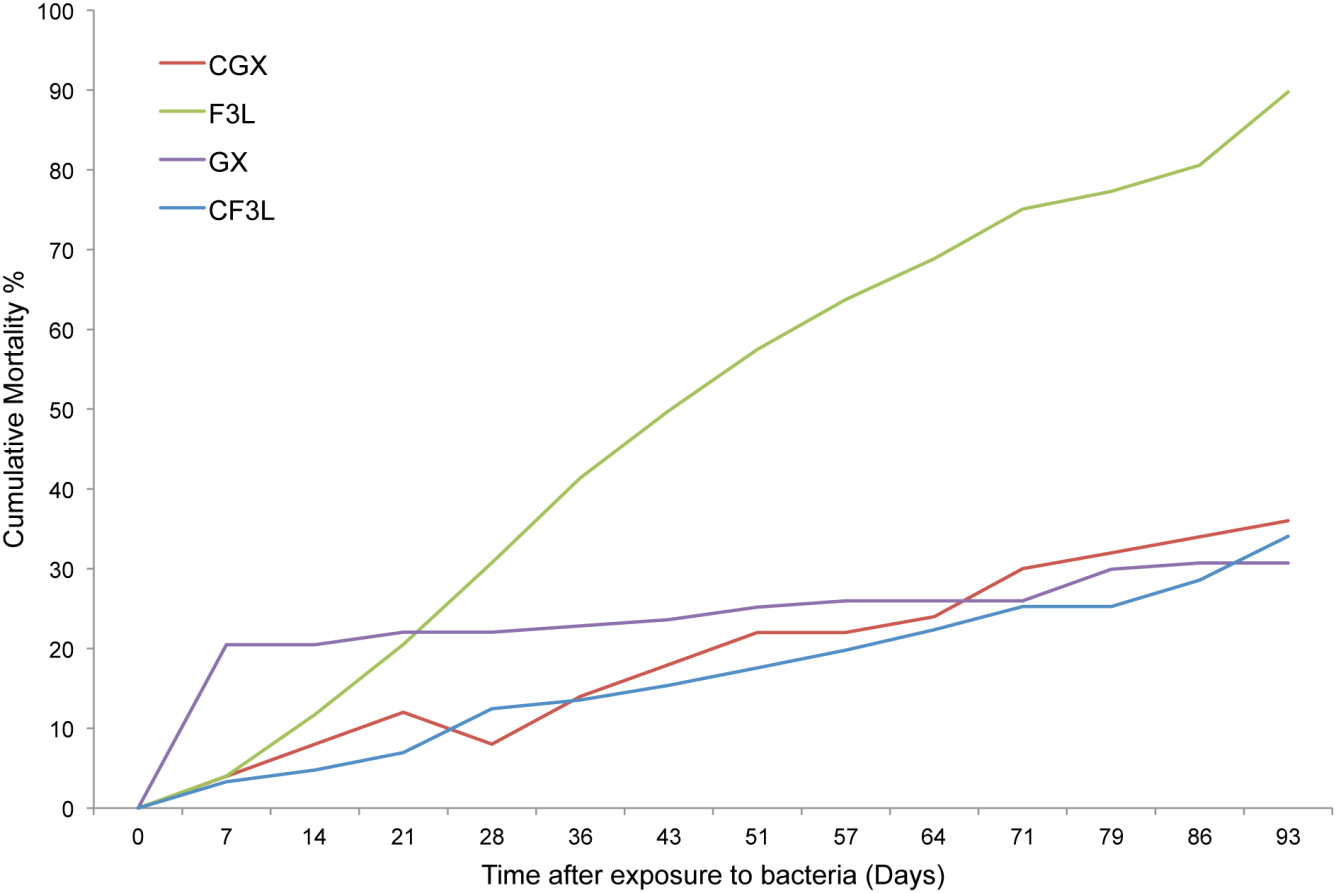
Mortality in resistant and susceptible oysters after challenge with *Roseovarius crassostreae*. Cumulative percent mortality in resistant GX and susceptible F3L oysters following bacterial challenge compared to mortality in non-challenged (CGX, CF3L) oysters. Arrows on the x-axis indicate the timepoints at which RNA was isolated for RNA-seq.

Oysters from the F3L family (susceptible to ROD) experienced a constant rate of mortality of about 7% per week after challenge with the bacterial pathogen *R. crassostreae*, reaching over 90% cumulative mortality by the end of the 93-day challenge period (Figure 1). The survival curve of the challenged susceptible F3L oysters was significantly different from all other groups (log-rank survival, *p*<0.01). At day 28, F3L had a significantly higher cumulative mortality than C3FL and GX (resistant to ROD), but not CGX control (*p*<0.01, Pearson’s chi-squared test with Bonferroni corrections). At day 93, F3L had a significantly higher cumulative mortality than GX, CGX, and C3FL (*p*<0.01). No significant differences in mortality were observed between unchallenged control oysters (CF3L and CGX) and oysters from the resistant challenged family GX at day 28 and day 93 after challenge. Challenged resistant oysters did not show any of the clinical signs of ROD, suggesting that the pathogen is eliminated rapidly and does not cause an active infection in these oysters. Oysters from the control resistant family CGX suffered a mortality event of unresolved origin between days 1 and 7 (20% cumulative percent mortality by day 7; Figure 1). Due to the potential confounding effect of this mortality event on gene expression at early time points after challenge, samples from CGX at days 1 and 5 were not included in the gene expression analysis.

### Oyster transcriptome assembly

From a total of 4.1×10^8^ Illumina GAIIx-sequenced cDNA reads of 108 bp, the final set consisted of 3.8×10^8^ reads of 94±5 bp after filtering and adaptor trimming. After the Trinity assembly of 374,029 contigs was filtered for contaminants, 356,237 contigs remained with a mean length of 440 bp and an N50 of 487 bp (link to assembly available at Text S1). A BLASTx search to the NCBI NR protein database led to annotation of 19.8% of the transcriptome. Of the total transcriptome, 22,934 contigs (16.3%) were at least 1 Kb in length. When the final set of processed reads (3.8×10^8^) were mapped to the transcriptome, 58% of the reads mapped to at least one alignment (Table 1).

**Table 1:** Assembly metrics, annotation information, and reads mapped for transcriptome assembly.

Number of contigs	356,237
Total span (bp)	156,920,694
Number of contigs >1Kb	22,934
Max Contig Length (bp)	16,256
Mean Contig Length (bp)	440
N50 (bp)	487
Number of contigs with BLAST hits*	70,621
% of contigs with BLAST hits*	19.8
% of reads mapped to transcriptome (bowtie [32])	58.13

*Contigs compared to NCBI’s non-redundant protein database using BLASTx, hits with e-value≤1e-06 retained.

### General patterns of gene expression in oysters in response to bacterial challenge

**Figure 2:**
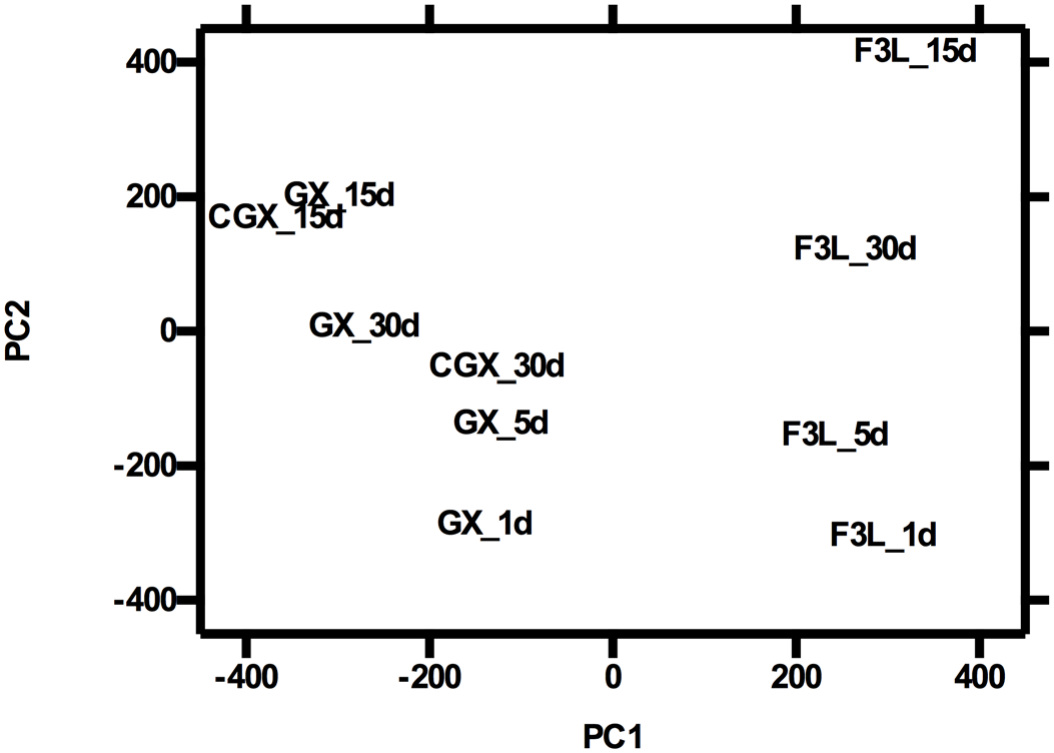
Principal components (PC) analysis of gene expression in resistant and susceptible oysters experimentally challenged with *Roseovarius crassostreae:* Spatial projection of PC1 and PC2. The Z-score centered log2-transformed RPKM for each transcript in challenged susceptible oysters at days 1, 5, 15, and 30 after challenge (F3L_1 to F3L_30), challenged resistant oysters at days 1 to 30 (GX_1 to GX_30), and unchallenged resistant oysters at days 15 and 30 (CGX_15, CGX_30) was used in the PCA. Data from unchallenged resistant oysters at days 1 and 5 were not included in the analysis due to the potential confounding effect of an unrelated mortality event observed before day 7. Gene expression in unchallenged susceptible oysters (CF3L) was not studied.

Principal components and heat map analyses were performed to evaluate general patterns of variation in gene expression between treatment groups. Principal components (PC) analysis showed that 93% of the variation in gene expression between groups is explained by 8 principal components, with 24%, 14%, and 12% of the variance explained by PC1, PC2, and PC3. The first PC separated treatments by family (F3L from GX), suggesting that the largest component of the variation (24%) in gene expression patterns can be attributed to genetic differences between the two families. The second component PC2 (explaining 14% of the variance) separated treatments by time after challenge (Figure 2). Gene expression patterns for GX-15d and CGX-15d, as well as GX-30 and CGX-30 clustered together in the projection of PC1 and PC2, showing relatively higher similarity between control and challenged resistant oysters at these time points. Based on similarity, comparisons of gene expression between resistant control and challenged oysters at days 15 and 30 were not included in further analyses of differential gene expression.

**Figure 3:**
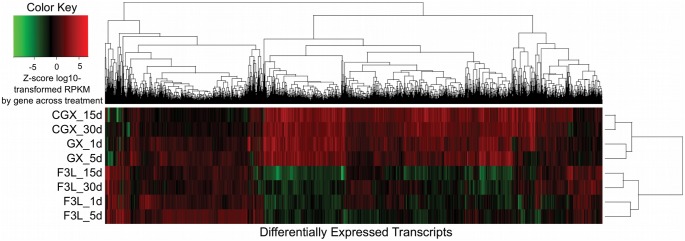
Heatmap of differentially expressed transcripts in resistant and susceptible oysters experimentally challenged with *Roseovarius crassostreae*. The Z-score centered log2-transformed RPKM for each transcript in each of eight sample groups is shown using a color scale. Genes are hierarchically clustered using Euclidean distance and complete linkage of the Z-score-transformed gene expression. Sample groups are clustered using the complete linkage Euclidean distance of the Spearman correlation of the Z-score-transformed gene expression.

Consistent with the results from the PCA, heat map cluster analysis showed two major clusters separating F3L and GX/CGX treatments, suggesting that a major portion of the variation in gene expression is due to genetic differences between the resistant and susceptible families (Figure 3). Within these major clusters, the following subclusters were detected that separated treatments within family based on time: GX 1 and 5d (designated GX_early), F3L 1 and 5d (F3L_early), F3L 15 and 30d (F3L_late), and CGX 15 and 30d (control) (Figure 3).

Based on: a) the unexplained mortality observed in the unchallenged resistant oysters by day 7, which precluded the use of the data from the control unchallenged resistant oysters (CGX) on days 1 and 5 oysters as controls for differential gene expression; and b) results from the PC (Figure 2) and heat map (Figure 3) analyses, which clustered samples first by family, and then by early and late time points, we decided to strengthen the statistical analysis of differential gene expression by considering data within each family at days 1 and 5 as replicates (GX_early, F3L_early). The same was done for the data from days 15 and 30 for the susceptible oysters (F3L_late). As a first step in identifying genes and processes differentially expressed in response to bacterial challenge, as well as genes that may be involved in disease resistance, differential gene expression of challenged resistant GX and susceptible F3L oysters at the early and late time points relative to the control unchallenged resistant oysters collected at 15 and 30 days (CGX_late) was determined.

**Figure 4:**
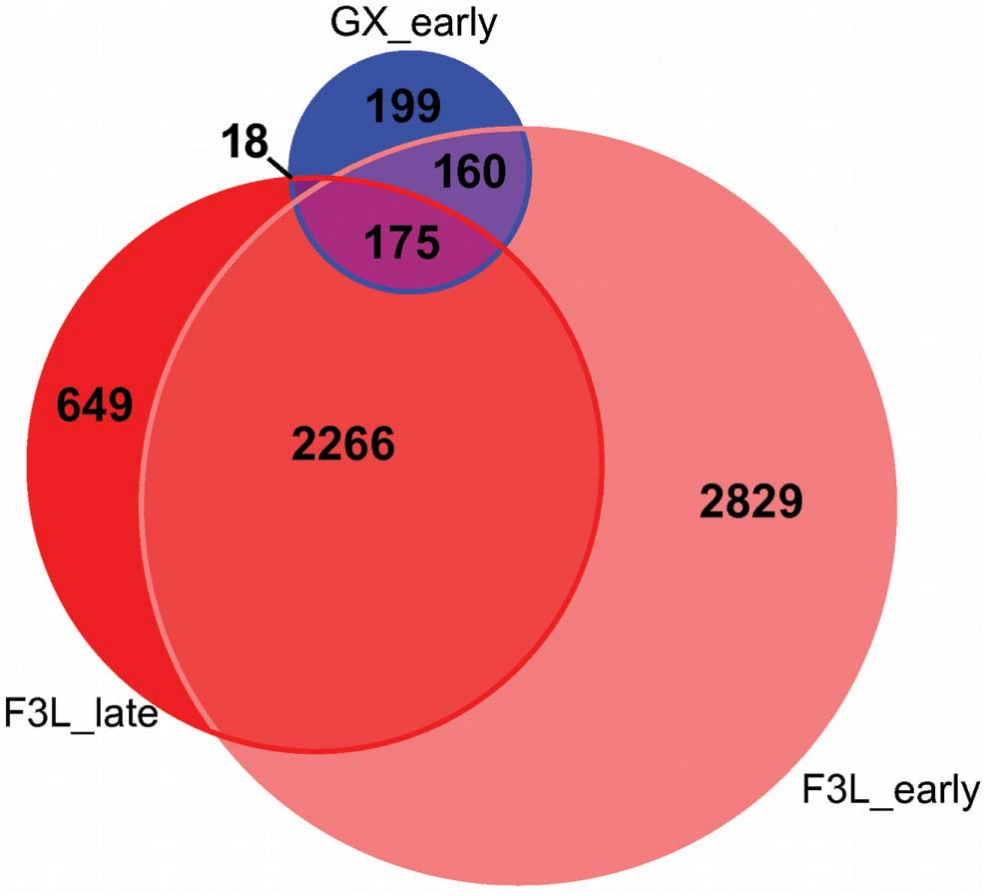
Differentially expressed transcripts in response to bacterial challenge shared and unique between resistant and susceptible oysters. Venn diagram of shared and unique differentiall expressed transcripts in GX_early (resistant family–days 1 and 5), F3L_early (susceptible – days 1 and 5), and F3L_late (susceptible – days 15 and 30) oysters after challenge with the bacterial pathogen *Roseovarius crassostreae*.

Of the 356,237 total transcripts tested for differential expression relative to CGX_late, 6,097 (1.7%) transcripts were differentially expressed in F3L_early and/or F3L_late, compared to only 552 (0.15%) transcripts differentially expressed in GX_early (Figure 4). This is consistent with the expectation that a relatively large component of the variation in gene expression is due to genetic differences between the oyster families. While differential gene expression in resistant (GX_early) oysters can be attributed to responses of oysters to challenge, as well as some temporal differences in gene expression, the larger amount of transcripts differentially expressed in susceptible F3L oysters is probably due to both differences in gene expression in response to challenge and to genetic differences between families.

### Enriched Gene Ontology terms and differentially expressed genes common to resistant and susceptible oysters in response to challenge with R. crassostreae

A gene ontology (GO) enrichment analysis was also performed to determine which biological processes were most highly represented (significantly enriched) amongst differentially expressed genes in each of the treatment groups. This approach to differential gene expression analysis should account for the variability in gene expression derived from the effect of time (day of sampling) while providing: a) a broad overview of the responses of oysters to challenge with *R. crassostreae* (differentially expressed genes and enriched GO terms observed in F3L and GX oysters in response to challenge); b) a list of genes potentially involved in disease susceptibility to ROD (genes differentially expressed in F3L, but not GX, in response to challenge); and c) a list of genes potentially involved in disease resistance to ROD (genes differentially expressed in GX but not detected in F3L, in response to challenge). Limitations of this approach will be addressed in the discussion section.

**Figure 5:**
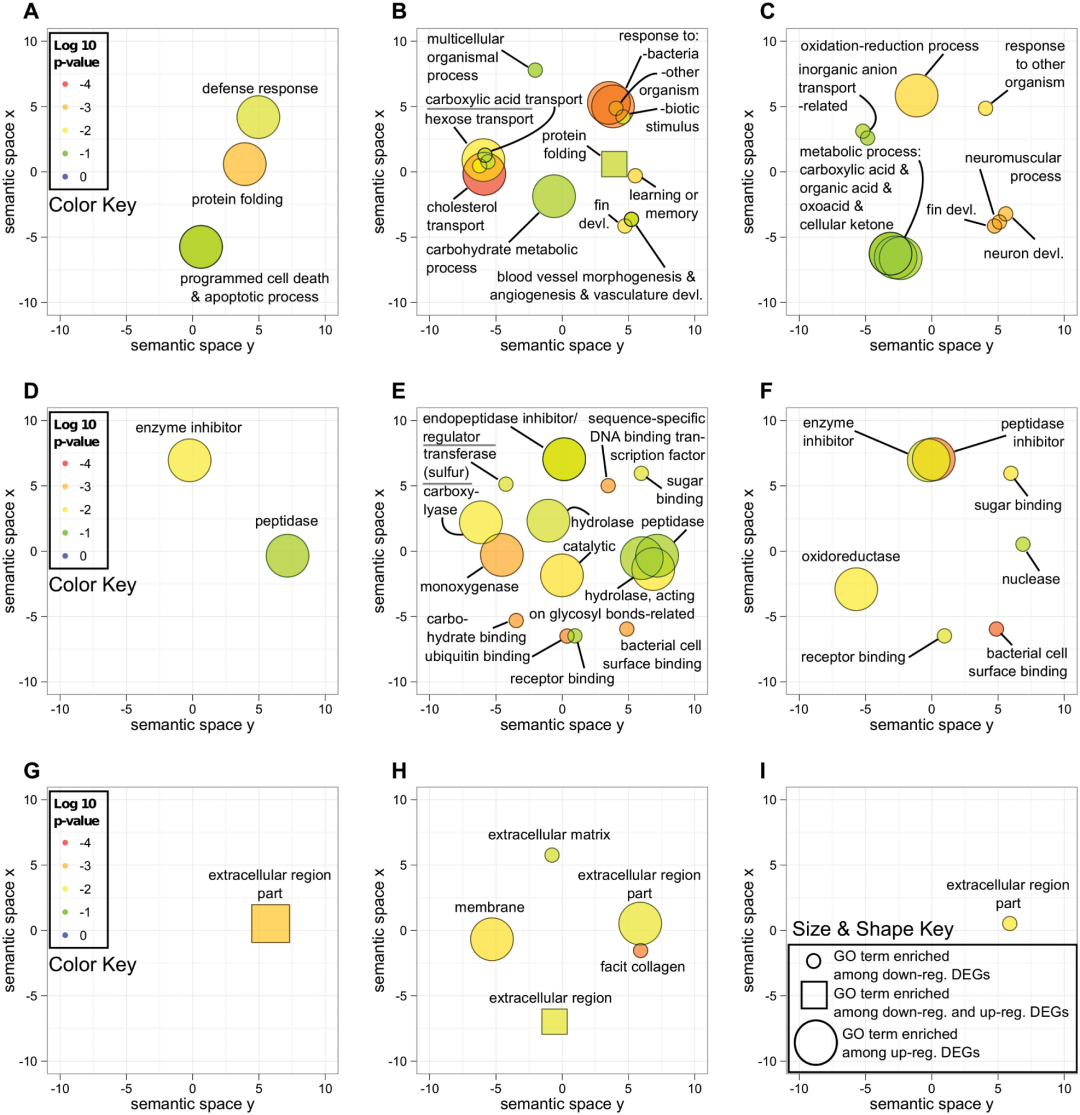
Functionally enriched Gene Ontology terms in the transcriptome of resistant and susceptible oysters in response to bacterial challenge. Functionally enriched Gene Ontology (GO) terms among differentially expressed transcripts in resistant oysters at 1 and 5 days after bacterial challenge (GX_early, A, D, G), susceptible oysters at 1 and 5 days after bacterial challenge (F3L_early, B, E, H), and susceptible oysters at 15 and 30 days after bacterial challenge (F3L_late, C, F, I) are displayed for biological processes (A–C), molecular function (D–F), and cellular component (G–I). Each GO term category is represented by a shape (circle or square) in the same x,y location in each of the graphs. The color of the shapes from cool (green) to warm (red) signifies increasing significance of enrichment as indicated in the color key. The size of shapes reflects whether the GO term is enriched among up-regulated DE transcripts (large) or down-regulated DE transcripts (small), while a GO term enriched among both up-regulated and down-regulated transcripts is represented by a square shape. Overlapping shapes corresponding to functionally similar categories have been labeled using a more general term, noted by the suffix “-related”.

Differentially expressed annotated genes and enriched Gene Ontology (GO) categories shared between resistant and susceptible oysters should provide a general view of the most conserved transcripts and molecular processes associated with host defenses to *R. crassostreae* in these 2 oyster families. Many of the genes diferentially expressed in among challenged oysters from both resistant GX and susceptible F3L families were associated with the gene ontology terms “defense response”, “defense response to bacterium”, “response to molecule of bacterial origin”, and “protein folding” (Figure 5A–C), as well as the related molecular functions “enzyme inhibitor activity”, “endopeptidase inhibitor activity”, “endopeptidase regulator activity”, and “peptidases” (Figure 5D–F). Examples of the most highly differentially expressed transcripts in response to bacterial challenge shared between resistant GX and susceptible F3L included several transcripts involved in immune recognition and signaling, such as C1q domain-containing (C1qDC) proteins, scavenger receptors cysteine-rich, c-type lectins, and dopamine-beta hydroxylase-like proteins. They also include several transcripts corresponding to the immune effectors serine protease inhibitors and a few annotated transcripts involved in detoxification, such as cytochrome p450 and glutathione S-transferase (Table 2).

**Table 2:** Annotated transcripts differentially expressed in both resistant (GX_early) and susceptible (F3L early and/or late) oysters in response to challenge with *R. crassostreae*.

Contig	Type ofDE (GX)	Type ofDE (F3L)	Annotation	Accession #
comp3607_c0_seq3	Up	Up	arylsulfatase a-like	XP_002607295
comp1799_c0_seq1	-	Down	c-type lectin	XP_002603342
comp10350_c0_seq1	Down	-	c-type lectin 2	XP_002603342
comp3136_c3_seq2	-	Down	c1q domain containing protein 1q11	CBX41660
comp887_c0_seq1	Up	Up	c1q domain containing protein 1q13	CBX41662
comp12483_c0_seq1	Down	Down	c1q domain containing protein 1q13	CBX41662
comp4668_c0_seq2	-	Down	c1q domain containing protein 1q40	CBX41689
comp1886_c0_seq5		Up	c1q domain containing protein 1q83	CBX41732
comp6091_c0_seq2	Down	Down	camp responsive element binding 2	AAU93879
comp1102_c0_seq3	Down	Down	collagen alpha-5 chain	XP_002595170
comp4943_c0_seq1	Up	Up	cytochrome family subfamily polypeptide 2-like	XP_002594971
comp13170_c1_seq3	Down	Down	deleted in malignant brain tumors 1	XP_002833280
comp7186_c1_seq4	Up	Up	dna damage-regulated autophagy modulator protein 2	NP_001230625
comp631_c0_seq1,2	Up	Up	dopamine beta hydroxylase-like	XP_002117561
comp1893_c0_seq1	Up	Up	dopamine beta hydroxylase-like	AAS92605
comp8625_c0_seq1	Up	Up	fatty acid synthase-like	ACZ55138
comp2451_c0_seq15	Up	Up	galactosamine (n-acetyl)-6-sulfate sulfatase-like	XP_002605064
several (>10) contigs	Down	Down	gtpase imap family members	XP_001920359, AAH96680
comp3498_c0_seq2	Up	Up	heat shock protein 60	ABN11936
comp5396_c0_seq2	Down	Down	melatonin receptor 1a	ADM73175
comp3971_c0_seq4	Down	Down	monocarboxylate transporter	XP_002573719
comp3971_c0_seq1	Down	Down	monocarboxylate transporter	XP_001606814
comp3971_c0_seq2	Down	Down	monocarboxylate transporter	EGI68511
comp11520_c0_seq1	Up	Up	nose resistant to fluoxetine family member (nrf-6)-like	XP_002600112
comp50794_c0_seq1	Down	Down	novel protein human megf11	EGW04058
comp30091_c0_seq2	Down	Down	nudt9	EGD73755
comp9303_c0_seq5	Up	Up	omega class glutathione s-transferase	CAD89618
comp11276_c0_seq3	Up	Up	polyketide synthase pks2	XP_002734101
comp25817_c0_seq1	Down	Down	protein tyrosine phosphatase	ACH42087
several contigs	Up	Up	scavenger receptor cysteine-rich protein	XP_001186391
comp2875_c0_seq2	Up	Up	serine protease	XP_002593726
comp3584_c0_seq1	Down	Up	serine protease inhibitor cvsi-1	Q30HU9
comp619_c0_seq1	Up	Up	serine protease inhibitor cvsi-2	B9A8D7
comp928_c0_seq1	Up	Up	serine protease inhibitor cvsi-2	B9A8D7
comp28180_c0_seq1	Down	Down	sushi-repeat-containing x-linked 2	XP_002932840
comp869_c0_seq2	Down	Down	x-box binding	XP_002732738

Contig number, direction of differential expression (up- or down-regulation), and name and accession number for the best BLASTx hits are shown.

Consistent with the extracellular nature of the infection by the bacterial pathogen *R. crasssotreae,* the most commonly differentially expressed transcripts in both resistant and susceptible oysters annotate to genes corresponding to the cellular component GO terms corresponding to membrane or extracellular regions (Figure 5G–I).

### Enriched Gene Ontology terms and differentially expressed genes unique to susceptible oysters in response to challenge with R. crassostreae

Annotated transcripts that are differentially expressed in challenged susceptible F3L (but not in challenged resistant GX oysters) relative to unchallenged CGX oysters, should include, among others, transcripts characteristic of the responses of oysters to an acute infection that may not be effective in removing the pathogen. These transcripts could be candidates for markers of disease susceptibility to ROD. Gene Ontology terms enriched in this group provide a general overview of the processes associated with the response of susceptible oysters to the bacterial pathogen. Many of the processes highly differentially regulated in susceptible F3L oysters (but not in resistant oysters) in response to challenge were related to metabolic functions, including hexose, carboxylic acid, and carbohydrate metabolic processes (Figure 5B,C), suggesting that infection with *R. crassostreae* may place a large metabolic demand on susceptible oysters. Examples of transcripts in these categories included several genes involved in detoxification, such as several transcripts for genes of the cytochrome p450 family (Table 3). Consistent with this, the terms “monooxygenase activity” (Fig. 5E) and “oxidoreductase activity” (Fig. 5F) were significantly enriched in susceptible oysters. The most significantly enriched F3L_early biological process term was “cholesterol transport” (Figure 5B), corresponding to epididymal secretory protein E1 (Table 3). Annotated transcripts showing the highest degree of differential expression in susceptible oysters, but not resistant oysters, included those coding for several heat shock proteins, several fibrinogen c domain-containing proteins, cadherin, legumain, vgd3, dermatopontin-2, and apextrin (Table 3).

**Table 3:** Top annotated transcripts differentially expressed in susceptible oysters at early and late timepoints after challenge with *R. crassostreae*.

Contig	logFC_1d	logFC_5d	logFC_15d	logFC_30d	Annotation	Accession #
comp1465_c0_seq5	−11.10	−11.00	-	-	adipose differentiation-related protein	XP_002595036
comp1612_c0_seq1	−10.62	−4.16	-	-	ankyrin unc44	XP_001190300
comp20450_c0_seq1	-	-	−10.12	−10.18	ankyrin unc44	XP_782809
comp781_c0_seq4	**-**	**-**	4.05	4.99	apextrin-like protein	AEK10749
comp6246_c1_seq1	2.83	4.62	-	-	cadherin- isoform h	BAD91058
comp4943_c0_seq1	**-**	**-**	3.27	4.55	cytochrome family subfamily polypeptide 2-like	XP_002594971
comp4943_c0_seq4	**-**	**-**	2.27	4.06	cytochrome p450 2b11	BAD02925
comp4943_c0_seq5	**-**	**-**	2.56	4.57	cytochrome p450 2g1-like	NP_001106451
comp4943_c0_seq6	**-**	**-**	3.54	4.74	cytochrome p450 2k	XP_002594509
comp1153_c0_seq7	−5.74	−7.45	**-**	**-**	dermatopontin 2	AAZ80787
comp1153_c0_seq5	−4.88	−6.67	**-**	**-**	dermatopontin 2	XP_001628981
comp1382_c0_seq1	**-**	**-**	7.21	8.66	dermatopontin 2	XP_001628981
comp211_c0_seq2	-	-	−9.54	−4.24	developmentally-regulated vdg3	ABB76764
comp211_c0_seq3	-	-	-	3.90	developmentally-regulated vdg3	ABB76764
comp211_c0_seq5	−12.84	−12.73	−8.48	−7.23	developmentally-regulated vdg3	ABB76764
comp7066_c0_seq1,2	-	-	-	4.14	developmentally-regulated vdg3	ABB76764
comp84_c0_seq2	3.74	5.64	-	-	developmentally-regulated vdg3	ABB76764
comp84_c0_seq3	2.82	4.73	-	-	developmentally-regulated vdg3	ABB76764
comp14053_c0_seq4	-	2.26	2.24	-	epididymal secretory protein e1 precursor	ACO09278
comp1891_c0_seq1	-	3.02	-	-	epididymal secretory protein e1 precursor	AAX61146
comp1891_c0_seq2	-	1.98	-	-	epididymal secretory protein e1 precursor	XP_003408814
comp1234_c1_seq4	−8.35	−6.77	-	-	fibrinogen c domain-containing protein 1-a-like	XP_003390678
comp3625_c0_seq2	−10.54	−5.97	-	-	fibrinogen c domain-containing protein 1-like	XP_003391179
comp3814_c0_seq1	**-**	**-**	−11.06	−6.66	fibrinogen-related protein	XP_002609404
comp9922_c0_seq7	5.53	2.52			heat shock 70 kda protein cognate 3	BAD15288
comp2543_c1_seq1	**-**	**-**	−11.71	−5.66	hemicentin 2-like	XP_002731765
comp7788_c0_seq2	−10.40	−5.31	-	-	hippocalcin-like protein 1	XP_001639635
comp6419_c0_seq3	−11.25	-	-	-	ing (mammalian inhibitor of growth) homolog family member (ing-3)-like	XP_002165512
comp8790_c1_seq1	-	-	−11.57	−5.16	legumain [Haliotis discus discus]	ABO26629
comp1637_c0_seq1	−5.38	−4.31	−2.86	−5.54	low affinity immunoglobulin epsilon fc receptor	NP_001138689
comp1890_c0_seq1	−10.44	−2.63	-	-	oncoprotein-induced transcript 3	XP_002595494
comp40_c0_seq9	6.11	8.14	-	-	period clock protein	ABM66066
comp1593_c0_seq2	−7.74	−6.47	-	-	proliferating cell nuclear antigen-like	XP_001631319
comp4352_c1_seq3	-	-	−10.54	−10.86	ring finger protein 145	AAH55485
comp11695_c0_seq2	5.11	4.01	7.25	6.17	tyrosine-protein kinase fer- partial	XP_002945302

Magnitude of differential expression in each treatment group is expressed as log_10_ fold change (logFC) over expression in the control group (CGX15/30d). The name and accession number for the best BLASTx hits are shown.

### Enriched Gene Ontology terms and differentially expressed genes unique to resistant oysters in response to challenge with R. crassostreae

Genes (annotations) differentially expressed in GX_early relative to unchallenged CGX_late that were not differentially expressed in susceptible oysters after bacterial challenge should include, among others, transcripts and processes contributing to host-defenses and disease resistance in the GX family. The biological process GO terms most significantly enriched among GX_early up-regulated transcripts and not present in susceptible oysters were the related terms “programmed cell death” and “apoptotic process,” corresponding to transcripts that annotated as inhibitor of apoptosis (IAP) proteins (Table 4). Other examples of differentially expressed annotated transcripts unique to GX_early included several transcripts that annotated to genes associated with the remodeling of the extracellular matrix (ECM), such as ADAMTS8 and furin, as well as several trancripts involved in immune recognition (scavenger receptor cysteine-rich), signaling (interleukin 17, rapunzel), and regulation of effector functions (arginase) (Table 4).

**Table 4:** Top annotated transcripts differentially expressed in resistant oysters at early time points after challenge with *R. crassostreae* (GX 1 and 5 days).

Contig	logFC_1d	logFC_5d	Annotation	Accession #
comp1506_c0_seq4	2.71	3.08	ADAMTS8	XP_002940685
comp4626_c0_seq4	3.34	-	alpha-ketoglutarate-dependent hypophosphite dioxygenase-like	XP_002944900
comp1285_c1_seq8	-	−3.90	arginase type-i-like, arginase-i	AEB70965
comp1285_c1_seq3	-	−3.54	arginase type-i-like, arginase-i	XP_002130834
comp5608_c0_seq1	-	−6.92	c-type lectin	ABB71672
comp24124_c0_seq1	-	4.49	ched related family member (ptr-19)	XP_002734100
comp5722_c1_seq1,2	-	−3.35	collagen alpha	XP_001512734
comp7972_c0_seq1	-	−8.09	cubilin	XP_002734392
comp7972_c0_seq4	-	−3.11	cubilin	XP_002612977
comp1788_c0_seq4	-	3.26	fibrinolytic enzyme	CAA64472
comp18756_c0_seq3	3.48	−	fibropellin ia	XP_002601363
comp6161_c0_seq11	-	2.48	furin	AAA49718
comp810_c1_seq1	-	−3.40	heat shock protein 22	ACU83231
comp18757_c0_seq1	-	−7.14	hla-b associated transcript 1	XP_003217350
comp2015_c0_seq13	−7.19	-	inhibitor of apoptosis (IAP)	AEB54799
comp2015_c0_seq24	3.95	-	inhibitor of apoptosis (IAP)	AEB54800
comp15440_c0_seq1	3.18	-	inhibitor of apoptosis (IAP)	XP_002426441
comp6837_c0_seq1	3.02	-	interleukin 17	A9XE49
comp3858_c0_seq5	3.63	-	isoleucyl-trna synthetase	NP_001090690
comp5396_c0_seq1	-	−3.01	melatonin receptor 1a	ADM73175
comp7137_c0_seq2	-	3.26	organic solute transporter subunit alpha	XP_002732822
comp39520_c0_seq1	-	−4.31	polyprotein	XP_002740782
comp24428_c0_seq1	−8.68	−8.64	rapunzel 5	NP_001103594
comp18902_c0_seq1	-	−3.43	rho gtpase	XP_002739105
comp1023_c0_seq2	-	2.62	scavenger receptor cysteine-rich	ACT53266
comp1023_c0_seq3	3.07	2.93	scavenger receptor cysteine-rich	XP_001622238
comp18756_c0_seq2	3.83	-	sushi repeat-containing	XP_002664481
comp25746_c0_seq4	-	−7.60	tenascin xb	XP_002741293
comp6161_c0_seq5	3.49	-	type 2 proinsulin processing endopeptidase	2206277A

Magnitude of differential expression in each treatment group is expressed as log_10_ fold change (logFC) over expression in the control group (CGX15/30d). The name and accession number for the best BLASTx hits are shown.

## Discussion

The two oyster families used in this study showed a dramatic difference in mortality to challenge with the bacterial pathogen *R. crassostreae,* causative agent of Roseovarius Oyster Disease (ROD) in juvenile oysters. While the susceptible oysters experienced constant levels of mortality due to ROD throughout the length of the challenge, oysters from the resistant family showed levels of mortality equal to the non-challenged oysters, even if they were continuously exposed to the pathogen through cohabitation with the susceptible oysters. We exploited these differences in mortality patterns, and ultimately differences in gene expression, to mine for genes and processes potentially involved in: 1) host-pathogen interactions in juvenile American oysters, and 2) disease resistance or susceptibility to ROD. Our analysis of the gene ontology terms most commonly represented (enriched) amongst the genes differentially expressed in challenged resistant and susceptible oysters relative to non-challenged oysters provides a broad view of the most conserved genes and processes involved in host responses of juvenile American oysters to *R. crassostreae*. We found that transcripts related to pathogen recognition, immune signaling and effector molecules, apoptosis, and detoxification were involved in the responses of the American oyster to bacterial challenge. In addition, we have identified several genes showing differential patterns of gene expression in either susceptible or resistant oysters in response to challenge, providing a useful foundation for the future identification of genes involved in disease resistance or susceptibility to ROD.

There are several limitations to this study that should be considered in the interpretation of the results. Although the levels of annotation achieved in this study (20% of the transcriptome) are comparable to the results of previous Illumina-generated transcriptome analyses in oysters (e.g. *C. gigas*, 16–23% annotated [48], [49]), our work should be viewed as an initial exploration of the most evolutionarily conserved aspects of the American oyster’s responses to *R. crassostreae* challenge. Furthermore, due to the limitations of the experimental design (differential gene expression in challenged F3L and GX was determined relative to that in unchallenged CGX oysters collected on days 15 and 30 after the start of the challenge), patterns of gene expression observed in this study could be due to either: a) true differences in gene expression between groups (for the most conserved genes); b) genetic differences between the families (transcripts corresponding to the same gene being identified as different genes in the assembly); and/or c) variability in gene expression between early and late time points (for the F3L_early and GX_early comparisons). Therefore, further work should be done in the future to validate the role of these genes in disease resistance/susceptibility to ROD and identify potential mechanisms of disease resistance. Constitutive and inducible differences in gene expression, as well as genetic differences between families are some of the mechanisms involved in disease resistance [13].

### Juvenile oyster responses to challenge with R. crassostreae

Differentially expressed annotated transcripts shared between resistant and susceptible oysters may provide insights into the most common immune responses of oysters to challenge with *R. crassostreae*. For both susceptible and resistant oysters, major immune responses to *R. crassostreae* included pathogen recognition, signaling, serine protease inhibition, detoxification, and apoptosis. Transcripts differentially expressed in both resistant and susceptible oysters in response to challenge annotating to genes involved in pathogen recognition included scavenger receptors cysteine-rich (SRs), C1qDC proteins, and c-type lectins [20]–[22], [50], [51]. Scavenger receptors are differentially expressed in oyster species in response to summer mortality [52], [53] and hypoxia [54]. Recently, an SR protein representing a novel class of scavenger receptor has been characterized in the scallop *Chlamys farreri* that is up-regulated by exposure to Pathogen Associated Molecular Patterns (PAMPs) like LPS, peptidoglycan and β-glucan and can bind LPS and peptidoglycan [55]. The role of C1qDC proteins as pathogen recognition receptors (PRRs) in molluscs has been solidified by a demonstration of the ability of a recombinant C1qDC protein from the scallop *Argopecten irradians* to bind PAMPs from diverse pathogens including gram-negative and gram-positive bacteria and fungi [56], [57]. Recently, a c-type lectin from *C. farreri* was shown to act as a PRR, binding LPS and β-glucan, and as an opsonin, enhancing the phagocytic capabilities of *C. farreri* hemocytes [58]. Interestingly, transcripts for fibrinogen-related proteins (FREPs) were differentially expressed in susceptible oysters but not in resistant oysters. This was an unexpected finding, since FREPs, which function in invertebrates in pathogen recognition, agglutination, and parasite resistance [59], have been shown in *B. glabrata* to contribute to resistance against the parasite *Schistosoma mansoni* [60]. A FREP in the bay scallop *Argopecten irradians* has agglutinating activity against chicken and human erythrocytes and bacteria and is up-regulated following challenge by gram-negative bacteria [61]. It is possible that FREPs expression in response to *R. crassostreae* may involve very early and acute up-regulation (before 24 h) or constitutive expression in resistant oysters.

Multiple dopamine beta-hydroxylase (DBH) transcripts were highly differentially expressed early in both resistant and susceptible oysters. DBH produce/modify catecholamines, which have been shown to modulate both the immune and stress response in vertebrates and invertebrates [62] including the scallop *C. farreri* [63], [64] and the Pacific oyster *C. gigas* [65], [66].

The responses of resistant and susceptible oysters to bacterial challenge also included several effectors of immunity responsible for minimizing or preventing damage caused by virulence factors from the pathogen. The second most highly up-regulated transcript at early time points for resistant oysters (also up-regulated in susceptible oysters) annotated as serine protease inhibitor-2 (Cvsi-2). Serine proteases are key virulence factors of many pathogens of bivalves, inhibiting phagocytosis in clams [66] and causing cytotoxicity of bivalve hemocytes [67], [68]. Serine protease inhibitor-1 (Cvsi-1), which was also highly up-regulated in susceptible oysters at early timepoints in this study, has been shown to have a role in the immune response of American oysters against the protozoan parasite *Perkinsus marinus*, likely by inhibiting the parasite’s major extracellular protease [69]. Moreover, polymorphism in the promoter of Cvsi-1 is associated with disease resistance to *P. marinus* [70]. We hypothesize that serine protease inhibitors may also play a role in neutralizing serine proteases released by *R. crassostreae*.

Transcripts differentially expressed in both resistant and susceptible oysters also included transcripts annotated as glutathione s-transferase, cytochrome p450, and multiple heat shock proteins, which are involved in detoxification and preventing oxidative damage. Glutathione s-transferase is an anti-oxidant and is up-regulated in hemocytes of Pacific oysters challenged with a pathogenic *Vibrio* sp. [17]. Although cytochrome p450s have been best studied in detoxification of xenobiotics in bivalves [71], they have also been implicated in the host defense responses of the flat oyster *Ostrea edulis* to the parasite *Bonamia ostreae* [72] and the Manila clam *Ruditapes phillipinarum* to *Vibrio* tapetis (the causative agent of Brown Ring Disease, a disease with clinical signs similar to ROD) [73]. Heat shock protein 60 is involved in xenobiotic detoxification and the stress response in oysters [74] and has an important role in immunity in mammals [75].

Another process involved in the responses of both the resistant and susceptible oysters to bacterial challenge was apoptosis, a form of programmed cell death that plays an important role in many processes, including immunity and development [76], [77]. In our study, inhibitor of apoptosis (IAP) and GTPase of the immunity-associated protein (GIMAP) transcripts were differentially expressed in both resistant and susceptible oysters. GIMAP proteins are important regulators of apoptosis [78]. Exposure of human monocytes to LPS induces the down-regulation of GIMAP, which may serve to promote the survival of monocytes by negatively regulating apoptosis [79]. We hypothesize that GIMAP proteins may serve an analogous function in oyster hemocytes. Apoptosis in general and IAP proteins in particular are associated with molluscan immunity [80], participating in the defense response of clams to *V. tapetis* [73], [81] and oysters to the protozoan parasite *P. marinus* [82], [83]. Four apoptosis-related genes, including IAP, were induced in Pacific oysters in response to challenge with the bacterial pathogen *V. anguillarum* [84]. Further work is needed to evaluate the potential role of IAP and GIMAP genes in hemocyte activation and survival.

### Potential genes involved in susceptibility to Roseovarius oyster disease

While resistant oysters appeared to be able to rapidly eliminate the pathogen, susceptible oysters suffered constant levels of morbidity and mortality throughout the challenge. Genes and processes activated in susceptible oysters in response to bacterial challenge and absent or present to a much lesser degree in resistant oysters may be used as indicators of an unsuccessful defense response and provide further insights on the molecular basis of disease susceptibility. Enrichment of transcripts corresponding to metabolic processes in susceptible oysters supports the hypothesis that a failed immune response against ROD places a large metabolic demand on these oysters, leading to mortality events [5], [6], [8]. Moreover, the unique enrichment in transcripts involved in detoxification (monooxygenase and oxidoreductase activities) in susceptible oysters could also be a reflection of the impact of ongoing acute infections in susceptible oysters, leading to upregulation of genes involved in minimizing the damage produced by the bacterial pathogen.

Other top genes differentially expressed in susceptible oysters but not in resistant oysters in our study that may warrant further investigation include epididymal secretory protein E1, cadherin, legumain, vdg3, dermatopontin 2, and apextrin. Epididymal secretory protein E1, also known as Niemann-Pick type C-2, facilitates cholesterol transport from lysosomes [85]. The up-regulation of epididymal secretory protein e1 uniquely in susceptible oysters at days 5 and 15 may represent lysomal turnover as an aspect of a continuous response to bacteria [86]. Cadherin is involved in cell adhesion [87], and legumain is a cysteine protease associated with response to bacteria [88], antigen processing [89], and ECM remodeling [90] in mammals. Transcripts annotating as dermatopontin-2, a shell matrix protein involved in cell adhesion and shell formation [91], [92] may be involved in bacterial encapsulation and conchiolin production (a hallmark clinical sign of ROD). Dermatopontin is strongly induced in amphioxus following bacterial challenge by gram-positive and gram-negative bacteria [93]. In the same bacterial challenge of amphioxus, another highly up-regulated gene was apextrin. Known primarily for its role in embryonal development [94], apextrin is a member of the membrane attack complex/perforin domain protein family [95] and is involved in innate antibacterial responses, possibly by sequestering or inactivating bacteria [96].

### Potential genes involved in resistance to Roseovarius oyster disease

Oysters from the resistant family did not show clinical signs of infection and suffered mortalities comparable to non-challenged oysters, suggesting that these oysters were able to eliminate the pathogen rapidly. ROD-resistant oysters responded to the bacterial pathogen *R. crassostreae* mainly by the differential expression of transcripts annotating to proteins that modify the extracellular matrix (ECM) (e.g. ADAMTS8), proteins that bind self or non-self ligands including pathogens (e.g. scavenger receptor cysteine-rich), stress proteins (e.g. HSP20, 60, and 70), and proteins involved in signaling (e.g. IL-17, cubilin, rapunzel). The up-regulation in resistant oysters of furin suggests the possible involvement of neuroendocrine signaling and/or host defense-relevant protein processing. Furin is involved in the processing of von Willebrand Factor, antimicrobial peptides in invertebrates, and certain matrix metalloproteinases; which in turn affect cell migration, differentiation, inflammation control, and the restructuring of the ECM [97]. The importance of ECM restructuring in the response of resistant oysters to bacterial challenge is corroborated by the up-regulation of a transcript annotating as ADAMTS8, a matrix metalloproteinase that is activated through cleavage by furin and likely participates in ECM proteolysis [98], as well as the differential expression of transcripts coding for tenascin-xb, an anti-adhesive glycoprotein involved in wound healing and matrix maturation [99].

The early response in resistant oysters also involved the pro-inflammatory mediator, interleukin 17 (IL-17), and the nitric oxide modulator, arginase. Members of the IL-17 family of cytokines induce the expression of antimicrobial proteins [100], and previous research suggests that IL-17 is an important mediator of the pro-inflammatory response in oysters [101]. Our results are consistent with the role of IL-17 in the immune response of oysters against bacterial infection and suggest a potential role in disease resistance to ROD. Arginases have been shown in macrophages to modulate the production of nitric oxide [102], which is an immune effector in the American oyster [103]. Using microarray technology, a transcript annotating as arginase was shown to increase rapidly after 6 h of heat stress in *C. gigas* [104]. The down-regulation of arginase in resistant oysters on day 5 may signal a down-regulation of the inflammation and stress response following a successful defense response.

In conclusion, this study shows that transcripts involved in processes such as pathogen recognition, extracellular matrix remodeling, detoxification, apoptosis, and regulation of the inflammatory (i.e. hemocytic infiltration) response may have an important role in the immune defenses of American oysters against *Roseovarius crassostreae,* the causative agent of Roseovarius Oyster Disease. This work represents the first deep characterization of the transcriptome of American oysters in response to a bacterial pathogen, providing many candidates genes and processes that should be targeted in the future characterization of mechanisms of resistance and susceptibility to this important bacterial disease of juvenile American oysters. The present study has also generated a pool of genes to be considered for further evaluation as candidate markers for advanced genotypic selection regimes for disease resistance in oysters.

## Supporting Information

Text S1 **Assembled transcriptome of American oysters in response to challenge with** ***Roseovarius crassostreae.***
(DOCX)
