# Supplementary material for: Transcriptome of American Oysters, Crassostrea virginica, in Response to Bacterial Challenge: Insights into Potential Mechanisms of Disease Resistance
Source: PLoS One. 2014 Aug 14;9(8):e105097. doi: 10.1371/journal.pone.0105097 (PMC4133350; doi:10.1371/journal.pone.0105097)
Supplement: Text S1 — Assembled transcriptome of American oysters in response to challenge with Roseovarius crassostreae. (DOCX) [file pone.0105097.s001.docx]

**Text S1. Assembled transcriptome of American oysters in response to challenge with *Roseovarius crassostreae.*** Processed reads were assembled using Trinity with default parameters. Accessible at FigShare (figshare.com) and at the Istrail’s laboratory webpage, http://www.brown.edu/Research/Istrail_Lab/rnaseq.php
